# Differences in the stress distribution in the distal femur between patellofemoral joint replacement and total knee replacement: a finite element study

**DOI:** 10.1186/1749-799X-7-28

**Published:** 2012-06-15

**Authors:** Hans-Peter W van Jonbergen, Bernardo Innocenti, Gian Luca Gervasi, Luc Labey, Nico Verdonschot

**Affiliations:** 1 Department of Orthopedic Surgery, Deventer Hospital, PO Box 5001, 7400 GC, Deventer, The Netherlands; 2 European Centre for Knee Research, Smith&Nephew, Technologielaan 11 Bis, 3001, Leuven, Belgium; 3 Department of Mechanical and Industrial Technology, University of Florence, Florence, Italy; 4 Department of Orthopaedic Surgery and Orthopaedic Research Laboratory, Radboud University Nijmegen Medical Center, PO Box 9101, 6500 HB, Nijmegen, The Netherlands

**Keywords:** Patellofemoral joint replacement, Knee prosthesis, Finite element analysis, Stress shielding, Squat movement

## Abstract

**Background:**

Patellofemoral joint replacement is a successful treatment option for isolated patellofemoral osteoarthritis. However, results of later conversion to total knee replacement may be compromised by periprosthetic bone loss. Previous clinical studies have demonstrated a decrease in distal femoral bone mineral density after patellofemoral joint replacement. It is unclear whether this is due to periprosthetic stress shielding. The main objective of the current study was to evaluate the stress shielding effect of prosthetic replacement with 2 different patellofemoral prosthetic designs and with a total knee prosthesis.

**Methods:**

We developed a finite element model of an intact patellofemoral joint, and finite element models of patellofemoral joint replacement with a Journey PFJ prosthesis, a Richards II prosthesis, and a Genesis II total knee prosthesis. For each of these 4 finite element models, the average Von Mises stress in 2 clinically relevant regions of interest were evaluated during a simulated squatting movement until 120 degrees of flexion.

**Results:**

During deep knee flexion, in the anterior region of interest, the average Von Mises stress with the Journey PFJ design was comparable to the physiological knee, while reduced by almost 25% for both the Richards II design and the Genesis II total knee joint replacement design. The average Von Mises stress in the supracondylar region of interest was similar for both patellofemoral prosthetic designs and the physiological model, with slightly lower stress for the Genesis II design.

**Conclusions:**

Patellofemoral joint replacement results in periprosthetic stress-shielding, although to a smaller degree than in total knee replacement. Specific patellofemoral prosthetic design properties may result in differences in femoral stress shielding.

## Background

Patellofemoral joint replacement is a successful treatment option for isolated patellofemoral osteoarthritis [1,2]. Only the patellofemoral joint is replaced, and the femorotibial compartments with cruciate ligaments and menisci are spared, which probably allows preservation of physiological femorotibial joint mechanics. The long-term outcomes of patellofemoral joint replacement are related to progression of femorotibial osteoarthritis and the need for conversion to total knee replacement [3]. Loss of distal femoral bone may compromise the results of such a conversion, and therefore needs to be prevented as much as possible. After total knee replacement, loss of bone occurs due to the stress shielding effect of the femoral component [4,5]. Although the femoral component of a patellofemoral prosthesis is smaller than in total knee replacement, it is unknown whether mechanically induced periprosthetic bone remodeling occurs following patellofemoral joint replacement.

Measurements of the periprosthetic bone mineral density (BMD) using dual-energy x-ray absorptiometry (DXA) demonstrated a 15% decrease in BMD behind the anterior flange of the femoral component during the first year after Richards II patellofemoral joint replacement [6], but it is not known whether this is due to stress shielding. Finite element analyses have been used extensively in the evaluation of prosthetic load, stress distribution in the bone and bone remodeling after total hip and knee replacement [7,8]. Some investigators have also used numerical models to calculate the stress distribution within the patellar components after patellofemoral joint replacement [9,10]. However, none of these models analyzed a loaded distal femur during a squat to investigate the effect of the femoral component on the stress distribution in the periprosthetic bone.

Our hypothesis was that patellofemoral joint replacement results in stress-shielding in the distal femur, but to a lesser extent than following total knee replacement. The objective of the current study was thus to investigate the effect of patellofemoral replacement on the expected stress distribution in the distal femur eventually leading to changes in bone density. For this purpose, the patellofemoral joint was modeled in a dynamic finite element knee model with and without a patellofemoral joint replacement. Furthermore, to investigate the effects of different patellofemoral joint replacement designs, we compared the Von Mises stresses between the Journey PFJ patellofemoral prosthesis, the Richards II patellofemoral prosthesis, and the Genesis II total knee prosthesis in two clinically relevant regions of interest.

## Methods

### Physiological knee joint geometry

**Figure 1:**
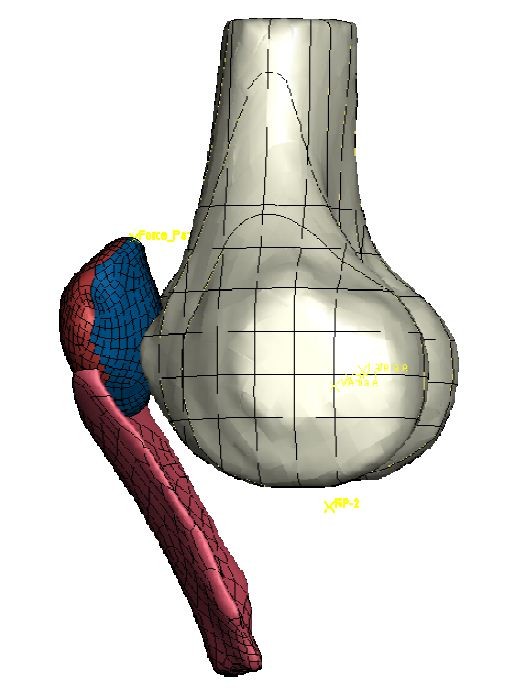
Physiological model geometry used in this study.

Magnetic Resonance (MR) imaging data (slice thickness 1.5 mm, pixel dimension 0.43 mm) from an intact human right knee cadaver specimen without known osteoarthritic changes were manually segmented using MIMICS 13 and 3-matic 4.2 software (Materialise, Belgium) and reconstructed into a three-dimensional model of the osseous and cartilaginous geometries of distal femur and patella with patellar tendon and insertion of the quadriceps tendon (Figure 1).

### Knee joint material properties

The trabecular bone of the distal femur was modeled as a homogenous isotropic linear elastic material (*E* = 300 MPa, Poisson ratio *v* = 0.30, density = 1 g/cm^3^) [11,12], while the cortical bone was modeled as an orthotropic 2 mm thick layer (*E*1 = 17900 MPa, *E*2 = 18800 MPa, *E*3 = 22800 MPa, G_23_ = 7110 MPa, G_31_ = 6580 MPa, G_12_ = 5710 MPa) [11-13]. The patella was modeled as a homogenous isotropic linear elastic material (*E* = 15000 MPa, Poisson ratio *v* = 0.30, density = 2 g/cm^3^), with a 2.5 mm thick layer of homogenous isotropic linear elastic cartilage (*E* = 5 MPa, Poisson ratio *v* = 0.46, density = 1 g/cm^3^) in contact with the femoral trochlea [12,14-16]. The friction coefficient between patella and trochlear groove was set at 0.001 based on experimental data [17]. The patellar tendon was modeled as an isotropic and hyperelastic material [16,18], with rubber-like mechanical behavior.

### Knee joint load and constraints

The simulated motion consisted of a 10s loaded full squat (one cycle), starting from 0° until a maximum flexion angle of 120°. These settings match the experimental kinematics simulations performed in a previous *in vitro* analysis on physiological cadaver legs [19-21]. The patella model was constrained by fixing the distal part of the patellar ligament and applying a quadriceps force distributed on the quadriceps insertion on the proximal surface of the patella, resulting in the patella moving along the trochlear surface of the femur [22]. The magnitude and direction of the quadriceps force as well as the three-dimensional kinematics of the femur were derived from the above mentioned tests on healthy, full leg cadaver specimens [21] and were applied using the Grood-Suntay coordinate system [23], using as origin the midpoint between the condylar center [19-21].

### Physiological knee joint finite element model

**Figure 2:**
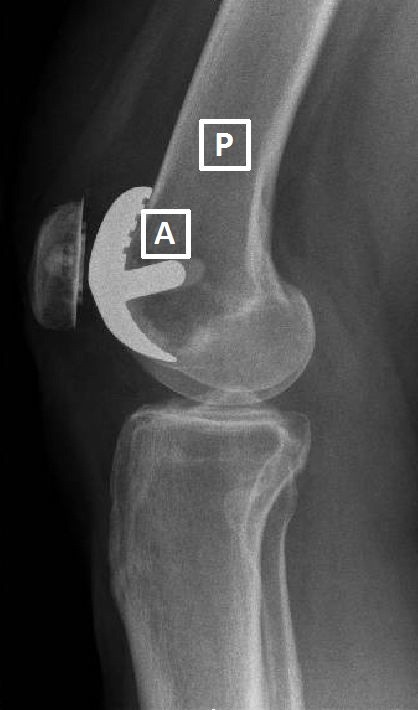
Location of anterior (A), and proximal (P) regions of interest (ROI) on a lateral radiograph of a right knee following Richards II patellofemoral joint replacement.

The three-dimensional models of femur, patella and patellar tendon were imported in commercially available finite element analysis software (Abaqus 6.8EF-1, Simulia, Dassault Systemes Paris, France). The models were meshed with 2 mm 6-noded triangle elements for the cortical and cartilage layers, and 2 mm 10-noded tetrahedral elements for the trabecular bone, patellar bone and patellar ligament. The number of elements for each component of the physiological knee model is given in Table 1. Two regions of interest (ROI) were defined in the femoral bone: an anterior and a proximal ROI. The location of the ROIs was defined to fit the same regions as used in a previous BMD analysis following patellofemoral joint replacement, thus allowing comparison with clinical data (Figure 2) [6]. The ROIs were 1 cm high in femoral proximo-distal direction and 1 cm long in the anteroposterior direction. They spanned the entire medio-lateral width. During the dynamic simulation the average Von Mises stress in each ROI was calculated.

**Table 1:** Number of elements for each component for the different finite element models

Finite element model	Femur	Patella	Patellar ligament	Femoral component	Patellar component
Physiological model	326737	93830	6914	/	/
Journey PFJ model	496078	26791	6914	18396	13346
Richards II model	318408	9375	6914	19522	4636
Genesis II model	160486	26791	6914	65503	13346

### Patellofemoral joint replacement finite element models

Three different joint replacement designs were considered in this study: Journey PFJ patellofemoral joint replacement (Smith&Nephew, Memphis, TN, USA), Richards II patellofemoral joint replacement (Smith&Nephew, Memphis, TN, USA), and Genesis II total knee replacement (Smith&Nephew, Memphis, TN, USA).

**Figure 3:**
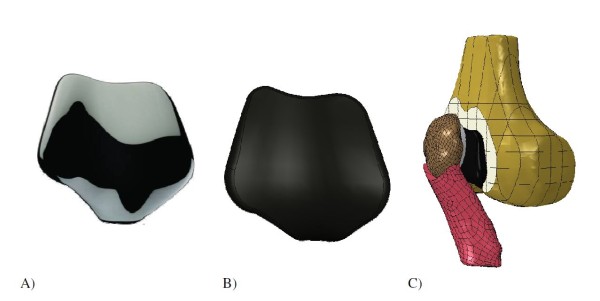
Journey PFJ patellofemoral prosthesis: A) Actual component, B) CAD file, C) Finite element model.

**Figure 4:**
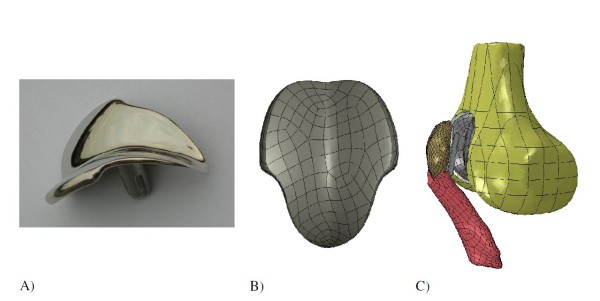
Richards II patellofemoral prosthesis: A) Actual component, B) CAD file, C) Finite element model.

The geometries of the Journey PFJ and Richards II prosthetic components were taken from CAD files provided by the manufacturer (Figures 3 and 4). The Journey PFJ femoral component (size medium) and ∅32 mm patellar component were incorporated in the knee model following the manufacturers’ instructions, and the Richards II femoral and patellar components (size small) were incorporated in the model using the previously described surgical technique [3]. The Journey PFJ femoral component (oxidized zirconium or Oxinium™) was modeled as an isotropic linear elastic material (*E* = 97905 MPa, Poisson ratio *v* = 0.3, density = 6.62 g/cm^3^) [24], while the Richards II femoral component (CoCr) was modeled as an isotropic linear elastic material (*E* = 240000 MPa, Poisson ratio *v* = 0.3) [25]. The UHMWPE patellar component was modeled as a non-linear elasto-plastic material (*E* = 684.65 MPa, Poisson ratio *v* = 0.45) [19,26,27]. A 2 mm cement layer with linear elastic material properties (*E* = 3000 MPa, Poisson ratio *v* = 0.3) was modeled between the prosthetic components and the cut bone surfaces [28,29]. The frictional coefficient between the UHMWPE patellar component and the Journey PFJ Oxinium femoral component was set at 0.04 based on experimental data [30], and we used a friction coefficient of 0.08 in the contact zone between the polyethylene patellar component and the Richards II CoCr femoral trochlear component [30]. The models were meshed with 2 mm 6-noded triangle elements for the cortical bone and cement layers, and 2 mm 10-noded tetrahedral elements for the cancellous bone, patellar bone, femoral component, patellar component and patellar ligament. The number of elements for each component of the patellofemoral joint replacement models is given in Table 1. We defined an anterior and a proximal ROI in the same positions and with the same dimensions as in the physiological model. During the dynamic simulation the average Von Mises stress in each ROI was calculated.

### Total knee replacement finite element model

**Figure 5:**
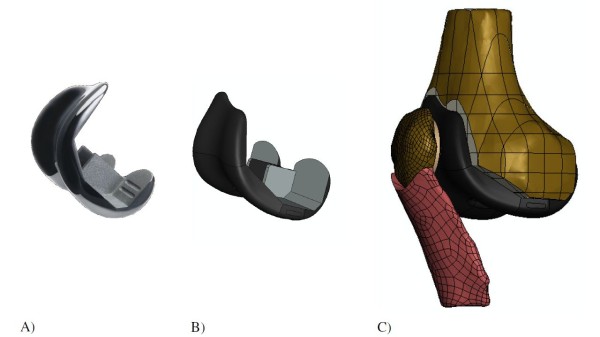
Genesis II total knee prosthesis: A) Actual component, B) CAD file, C) Finite element model.

The geometries of the Genesis II total knee prosthetic components were taken from CAD files provided by the manufacturer (Figure 5). The Genesis II posterior stabilized total knee femoral component (size 5) and ∅32 mm patellar component were incorporated in the knee model following the manufacturers’ instructions. The Genesis II total knee prosthesis was chosen for the total knee model because the geometry of the articular surface of the femoral component of this prosthesis is exactly the same as the geometry of the articular surface of the Journey PFJ femoral component. The material properties of the femoral component (Oxinium™), patellar component and cement were the same as in the Journey PFJ model. Also, the contact, friction and mesh properties were the same as in the Journey PFJ model. The number of elements for each component of the Genesis II model is given in Table 1. We defined an anterior and a proximal ROI in the same positions and with the same dimensions as in the physiological model. During the dynamic simulation the average Von Mises stress in each ROI was calculated.

### Convergence analysis and validation

A convergence analysis was performed to evaluate the effect of mesh quality and density changes on the calculated stress distributions. We created 5 mesh files of different uniform element sizes ranging from 4 to 2 mm. For each mesh size, the contact force, contact area, and contact pressure were calculated as a function of flexion angle. With a sequence of finer meshes, i.e. increasing the mesh density, the curves converged to the same solutions. Similarly, we ensured that models with different numbers of elements converged.

**Figure 6:**
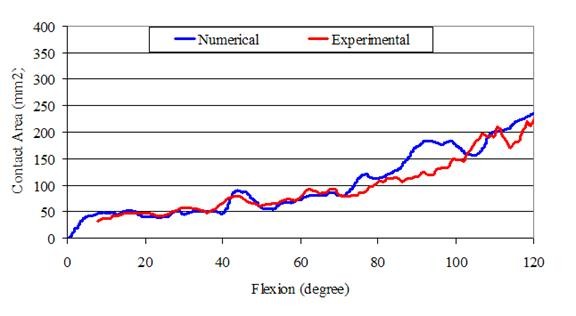
Comparison of contact area versus flexion angle for numerical model and knee kinematics simulator experiment.

We compared the numerically determined contact area with the contact area obtained using contact pressure sensors in a knee kinematics simulator experiment using the same specimen, with the same load and boundary conditions as those used for the numerical simulation [31]. The results confirmed the validity of the model as shown in Figure 6. Moreover, the values found for patellofemoral peak force were in agreement with values reported in the literature [22,32,33].

## Results

**Figure 7:**
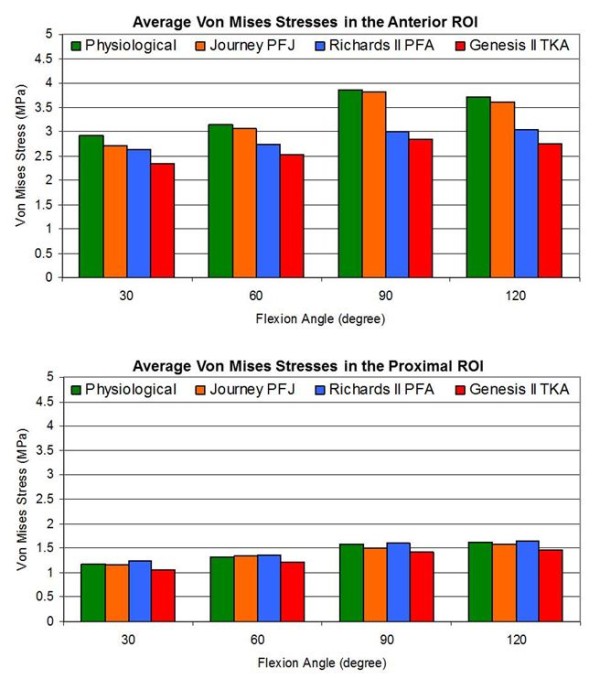
Average Von Mises stress in the anterior and proximal ROI in the 4 models for discrete flexion angles of simulated squat motion.

Figure 7 shows the predicted average Von Mises stress in the anterior and proximal ROI for the 4 models at discrete flexion angles of simulated squat motion. Overall, the average Von Mises stress in both ROIs increased with the flexion angle. Maximum stresses during squat were reached at 90° flexion angle (2.8-3.8 MPa for the anterior ROI, and 1.4-1.6 MPa for the proximal ROI).

Mean stresses in the proximal ROI were similar for both patellofemoral joint replacement designs and the physiological model, and slightly lower for the Genesis II total knee prosthetic design. Between 80° and 120° flexion, anterior ROI bone stresses for the Journey PFJ design were comparable to the physiological knee, while reduced by almost 25% for both the Richards II patellofemoral joint and Genesis II total knee joint replacement designs.

## Discussion

The aim of the current study was to evaluate the effect of patellofemoral joint replacement on the expected stress-distribution in the distal femur leading to bone density changes. We modeled a patellofemoral joint in a finite element knee model with and without a patellofemoral joint replacement. In order to investigate the effects of different patellofemoral replacement designs, we also compared the Von Mises stress distribution induced by the patellofemoral load between the Journey PFJ patellofemoral prosthesis, the Richards II patellofemoral prosthesis, and the Genesis II total knee prosthesis.

In summary, our dynamic finite element modeling demonstrated comparable Von Mises stress in the anterior region of interest for the Journey PFJ design and the physiological knee. The Richards II design demonstrated lower average Von Mises stress in the anterior region of interest compared to the physiological knee, and the Genesis II total knee design demonstrated the lowest average Von Mises stress. The average Von Mises stress in the proximal region of interest were similar for both patellofemoral designs and the physiological model, with slightly lower average Von Mises stress for the Genesis II total knee prosthesis.

Stress shielding behind the anterior flange of a patellofemoral prosthesis may result in mechanically induced bone remodeling with resulting decrease in BMD. Our Journey PFJ model predicted no significant stress shielding, implying that the physiological strains are maintained. However, the Richards II model predicted a reduction in average Von Mises stress compared to the physiological model. This is in agreement with the recently reported results of clinical dual-energy X-ray absorptiometry (DXA) measurements obtained in 14 patients [6]. In this prospective 1-year DXA study a 15% decrease in BMD was found behind the anterior flange of the Richards type II patellofemoral prosthesis.

The observed differences in the stress shielding effect between the Richards II and Journey PFJ prostheses may result from differences in both material and geometrical properties. The Journey PFJ prosthesis is considered a third generation patellofemoral prosthesis. The implant is characterized by a broad anatomical trochlear component made of oxidized zirconium (Oxinium™) which has a coefficient of friction that is half that of CoCr. There are 4 small fixation pegs on the posterior aspect of the femoral component. In contrast, the Richards II prosthesis is a first-generation CoCr prosthesis. The non-anatomic trochlear component is highly constrained with a deep central groove, and the polyethylene patellar component has a longitudinal ridge. In addition, the femoral component has an approximately 2 cm long central fixation peg for added stability. This long central fixation peg may in part explain the pronounced stress shielding effect observed at 90 degrees of flexion, since the patella contacts the femoral component below the position of the peg. Furthermore, the Journey PFJ femoral component is shorter in the sagittal plane than the Richards II component. As a result, in deep flexion the patellar button contacts the native femoral surface beyond 90 degrees of flexion while in the Richards II design there is still contact between the femoral component and the patellar button.

After total knee replacement, the BMD of the distal femur decreases by 16–36% within one year because of the femoral component’s stress-shielding effect [34-40]. Finite element analyses have shown that with a bonded femoral component, the predicted long-term bone loss would occur at the most distal part of the femur and behind the anterior part of the prosthesis [4,5]. This is in agreement with our findings.

Only one previous study reported results from a finite element knee model in patellofemoral joint replacement [10]. Using the geometries of the Low Contact Stress (LCS), Vanguard, and Scout patellofemoral prostheses, a three-dimensional finite element model of each particular design was created. Stress distributions within the patellar components were calculated for three common daily activities: walking, ascending stairs, and rising from a chair. Static loading was applied with 420 N patellofemoral joint reaction force in 15° of flexion (walking gait), 1760 N in 45° of flexion (stair ascent), and 1950 N in 90° of flexion (chair rise). Based on these models, the authors concluded that different patellofemoral implant design geometries influence the polymer stress within the patellar component. To minimize contact and delamination stresses, they further stated that contemporary designs should employ a broad trochlear groove to maximize congruent patellar component contact. No attempt was, however, made to evaluate for altered stress distributions in the periprosthetic distal femur. In an experimental setup using tri-axial strain gauges in synthetic femurs before and after Journey PFJ patellofemoral joint replacement, Meireles et al. determined the strain shielding effect in the distal femur [41]. A strain shielding effect was found with static loading of the patellofemoral joint in 12, 50, and 90 degrees of flexion. The results showed a reduction in strain in the medial and distal regions of the femur when deep bending occurred, with higher values of strain in the anterior region proximal to the prosthesis. This distal diaphyseal increase in strain has also been noted in finite element models with total knee replacement [5].

The present study is the first to specifically evaluate for the stress-shielding effect of a patellofemoral prosthesis using finite element modeling. However, correct interpretation of results obtained from numerical models requires careful consideration of several important issues [42]. We employed a numerical model that has been both verified and validated using the same procedure as described by Catani et al. [31]. Moreover, the biological findings based on clinical DXA measurements behind the anterior flange of the Richards II patellofemoral prosthesis [6] match the findings of this finite element analysis. Of course, validation will only be complete by adding clinical DXA measurements in the same ROIs for the other types of implant that were considered in this simulation. This information is not available yet but it is the subject of an ongoing study.

Finite element modeling is, however, subject to limitations due to inherent uncertainties concerning geometry, load situation, and material properties. We performed our modeling using a dynamic loaded squat instead of a gait cycle, which is a more frequent activity. Squatting is a motor task which produces higher joint contact forces and therefore higher stresses and strains in the bone compared to gait [43,44]. Moreover, as squatting gives rise to higher stresses, it should also induce a larger difference in stress between the physiological and replaced knee. Finally, the analysis of a squat, up to 120°, was preferred since it reaches a larger range of motion compared to gait which is usually limited to 60° of flexion [45]. Nevertheless, analysis of other activities will probably lead to similar relative results but in a smaller range of knee flexion and with smaller differences in periprosthetic Von Mises stress values. Although we employed a dynamic simulation, we did not consider viscoelastic properties of the materials. Because the knee flexion from 0° to 120° was performed in 10s, the time dependent effect was assumed negligible. Furthermore, in the simulated activity, the patellar tendon was always loaded during the entire cycle and no unloaded situation or hysteresis was present nor simulated.

A further limitation of our study was that we evaluated the stress-shielding effect of three different joint replacement designs from one manufacturer (Smith&Nephew, Memphis, TN, USA). Prosthetic designs from other manufacturers differ with respect to material and design properties. Inclusion of other designs in our FE modeling would probably have shown differences in the amount of stress shielding, and not including these designs therefore limits the scope of our study.

Finally, we did not consider potential failure of the bone-implant interface due to elevated stress levels. Although less stress shielding means better load transfer to the bone, which is desirable, it may also imply higher loads in the interface region and an increased risk for failure of the fixation that was not considered in the present analysis. However, this limitation is not clinically relevant as loosening of cemented femoral components is not an issue with isolated patellofemoral joint replacement [3].

## Conclusions

Dynamic finite element analyses of knee models with a patellofemoral joint replacement predict a decrease in average bone stress behind the anterior flange of the femoral component. This reduction was more pronounced in the Richards II design than in the Journey PFJ design, and may be related to specific design properties.

## Competing interests

Both Bernardo Innocenti and Luc Labey wish to note that they are employees of the European Centre for Knee Research, Smith&Nephew, Belgium.

## Authors’ contributions

All the authors participated in the design of the study. BI, GLG, and LL designed the computer model and performed the finite element analysis. All authors helped to draft the manuscript. All authors read and approved the final manuscript.

## References

[B1] van Jonbergen HPW Poolman RW van Kampen A Isolated patellofemoral osteoarthritis: a systematic review of treatment options using the GRADE approach Acta Orthop 2010 81 199 205 10.3109/17453671003628756 20175647 PMC2852157

[B2] Gupta RR Zywiel MG Leadbetter WB Bonutti P Mont MA Scientific evidence for the use of modern patellofemoral arthroplasty Expert Rev Med Devices 2010 7 51 66 10.1586/erd.09.53 20021240

[B3] van Jonbergen HPW Werkman DM Barnaart LF van Kampen A Long-term outcomes of patellofemoral arthroplasty J Arthroplasty 2010 25 1066 1071 10.1016/j.arth.2009.08.023 20056375

[B4] Tissakht M Ahmed AM Chan KC Calculated stress-shielding in the distal femur after total knee replacement corresponds to the reported location of bone loss J Orthop Res 1996 14 778 785 10.1002/jor.1100140515 8893772

[B5] van Lenthe GH de Waal Malefijt MC Huiskes R Stress shielding after total knee replacement may cause bone resorption in the distal femur J Bone Joint Surg Br 1997 79 117 122 10.1302/0301-620X.79B1.6808 9020459

[B6] van Jonbergen HPW Koster K Labey L Innocenti B van Kampen A Distal femoral bone mineral density decreases following patellofemoral arthroplasty: 1-year follow-up study of 14 patients BMC Musculoskelet Disord 2010 11 74 10.1186/1471-2474-11-74 20406477 PMC2864205

[B7] Qian JG Song YW Tang X Zhang S Examination of femoral-neck structure using finite element model and bone mineral density using dual-energy X-ray absorptiometry Clin Biomech 2009 24 47 52 10.1016/j.clinbiomech.2008.09.007 18980785

[B8] Zelle J Van der Zanden AC De Waal MM Verdonschot N Biomechanical analysis of posterior cruciate ligament retaining high-flexion total knee arthroplasty Clin Biomech 2009 24 842 849 10.1016/j.clinbiomech.2009.08.004 19733944

[B9] Najarian S Rostami M Rezaei T Hamza MH Biomechanical analysis of patellofemoral joint prosthesis using finite element method Proceedings of the Second IASTED International Conference on BioMechanics: 7-9 September 2005; Benidorm, Spain 2005 ACTA Press, Calgary 265 268

[B10] Morra EA Greenwald AS Patellofemoral replacement polymer stress during daily activities: a finite element study J Bone Joint Surg Am 2006 88 Suppl. 4 213 216 17142450 10.2106/JBJS.F.00585

[B11] Heegaard JH Leyvraz PF Hovey CB A computer model to simulate patellar biomechanics following total knee replacement: the effects of femoral component alignment Clin Biomech 2001 16 415 423 10.1016/S0268-0033(01)00020-1 11390049

[B12] Beillas P Papaioannou G Tashman S Yang KH A new method to investigate in vivo knee behavior using a finite element model of the lower limb J Biomech 2004 37 1019 1030 10.1016/j.jbiomech.2003.11.022 15165872

[B13] Taylor WR Roland E Ploeg H Hertig D Klabunde R Warner MD Hobatho MC Rakotomanana L Clift SE Determination of orthotropic bone elastic constants using FEA and modal analysis J Biomech 2002 35 767 773 10.1016/S0021-9290(02)00022-2 12020996

[B14] Iranpour F Merican AM Amis AA Cobb JP The width:thickness ratio of the patella: an aid in knee arthroplasty Clin Orthop 2008 466 1198 1203 10.1007/s11999-008-0130-x 18330664 PMC2311467

[B15] Pena E Calvo B Martinez MA Doblare M A three-dimensional finite element analysis of the combined behavior of ligaments and menisci in the healthy human knee joint J Biomech 2006 39 1686 1701 10.1016/j.jbiomech.2005.04.030 15993414

[B16] Pena E Calvo B Martinez MA Palanca D Doblare M Finite element analysis of the effect of meniscal tears and meniscectomies on human knee biomechanics Clin Biomech 2005 20 498 507 10.1016/j.clinbiomech.2005.01.009 15836937

[B17] Besier TF Gold GE Beaupre GS Delp SL A modeling framework to estimate patellofemoral joint cartilage stress in vivo Med Sci Sports Exerc 2005 37 1924 1930 10.1249/01.mss.0000176686.18683.64 16286863

[B18] Weiss JA Gardiner JC Computational modeling of ligament mechanics Crit Rev Biomed Eng 2001 29 303 371 10.1615/CritRevBiomedEng.v29.i3.20 11730098

[B19] Innocenti B Truyens E Labey L Wong P Victor J Bellemans J Can medio-lateral baseplate position and load sharing induce asymptomatic local bone resorption of the proximal tibia? A finite element study J Orthop Surg Res 2009 4 26 10.1186/1749-799X-4-26 19615054 PMC2718929

[B20] Victor J Van Glabbeek F Vander Sloten J Parizel PM Somville J Bellemans J An experimental model for kinematic analysis of the knee J Bone Joint Surg Am 2009 91 Suppl. 6 150 163 19884423 10.2106/JBJS.I.00498

[B21] Victor J Labey L Wong P Innocenti B Bellemans J The influence of muscle load on tibiofemoral knee kinematics J Orthop Res 2010 28 419 428 19890990 10.1002/jor.21019

[B22] Sharma A Leszko F Komistek RD Scuderi GR Cates HE Liu F In vivo patellofemoral forces in high flexion total knee arthroplasty J Biomech 2008 41 642 648 10.1016/j.jbiomech.2007.09.027 17983624

[B23] Grood ES Suntay WJ A joint coordinate system for the clinical description of three-dimensional motions: application to the knee J Biomech Eng 1983 105 136 144 10.1115/1.3138397 6865355

[B24] Wah Chang Zirconium Products: Technical Data Sheet 2003 An Allegheny Technologies Company

[B25] Davis JR Handbook of Materials for Medical Devices 2003 Materials Park, Ohio: ASM International

[B26] Godest AC Beaugonin M Haug E Taylor M Gregson PJ Simulation of a knee joint replacement during a gait cycle using explicit finite element analysis J Biomech 2002 35 267 275 10.1016/S0021-9290(01)00179-8 11784545

[B27] Halloran JP Petrella AJ Rullkoetter PJ Explicit finite element modeling of total knee replacement mechanics J Biomech 2005 38 323 331 10.1016/j.jbiomech.2004.02.046 15598460

[B28] Vaninbroukx M Labey L Innocenti B Bellemans J Cementing the femoral component in total knee arthroplasty: which technique is the best? Knee 2009 16 265 268 10.1016/j.knee.2008.11.015 19138857

[B29] Janssen D Mann KA Verdonschot N Micro-mechanical modeling of the cement-bone interface: the effect of friction, morphology and material properties on the micromechanical response J Biomech 2008 41 3158 3163 10.1016/j.jbiomech.2008.08.020 18848699 PMC2613656

[B30] Poggie RA Wert JJ Mishra AK Davidson JA Denton R, Keshavan MK Friction and wear characteristics of UHMWPE in reciprocating sliding contact with Co-Cr, Ti-6A1-4V, and Zirconia implant bearing surfaces Wear and Friction of Elastomers, ASTM STP 1145 1992 Philadelphia: American Society for Testing and Materials 65 81

[B31] Catani F Innocenti B Belvedere C Labey L Ensini A Leardini A The Mark Coventry Award: Articular contact estimation in TKA using in vivo kinematics and finite element analysis Clin Orthop Relat Res 2010 468 19 28 10.1007/s11999-009-0941-4 19548042 PMC2795837

[B32] Wretenberg P Feng Y Arborelius UP High- and low-bar squatting techniques during weight-training Med Sci Sports Exerc 1996 28 218 224 10.1097/00005768-199602000-00010 8775157

[B33] Innocenti B Pianigiani S Labey L Victor J Bellemans J Contact forces in several TKA designs during squatting: A numerical sensitivity analysis J Biomech 2011 44 1573 1581 10.1016/j.jbiomech.2011.02.081 21435645

[B34] Abu-Rajab RB Watson WS Walker B Roberts J Gallacher SJ Meek RM Peri-prosthetic bone mineral density after total knee arthroplasty. Cemented versus cementless fixation J Bone Joint Surg Br 2006 88 606 613 10.1302/0301-620X.88B5.16893 16645105

[B35] Karbowski A Schwitalle M Eckardt A Heine J Periprosthetic bone remodelling after total knee arthroplasty: early assessment by dual energy X-ray absorptiometry Arch Orthop Trauma Surg 1999 119 324 326 10.1007/s004020050419 10447632

[B36] Liu TK Yang RS Chieng PU Shee BW Periprosthetic bone mineral density of the distal femur after total knee arthroplasty Int Orthop 1995 19 346 351 8567149 10.1007/BF00178346

[B37] Petersen MM Olsen C Lauritzen JB Lund B Changes in bone mineral density of the distal femur following uncemented total knee arthroplasty J Arthroplasty 1995 10 7 11 7730833 10.1016/s0883-5403(05)80094-4

[B38] Soininvaara TA Miettinen HJ Jurvelin JS Suomalainen OT Alhava EM Kroger HP Periprosthetic femoral bone loss after total knee arthroplasty: 1-year follow-up study of 69 patients Knee 2004 11 297 302 10.1016/j.knee.2003.09.006 15261216

[B39] Spittlehouse AJ Getty CJ Eastell R Measurement of bone mineral density by dual-energy X-ray absorptiometry around an uncemented knee prosthesis J Arthroplasty 1999 14 957 963 10.1016/S0883-5403(99)90010-4 10614887

[B40] van Loon CJ Oyen WJ de Waal Malefijt MC Verdonschot N Distal femoral bone mineral density after total knee arthroplasty: a comparison with general bone mineral density Arch Orthop Trauma Surg 2001 121 282 285 10.1007/s004020000232 11409560

[B41] Meireles S Completo A Antonio SJ Flores P Strain shielding in distal femur after patellofemoral arthroplasty under different activity conditions J Biomech 2010 43 477 484 10.1016/j.jbiomech.2009.09.048 20004900

[B42] Viceconti M Olsen S Nolte LP Burton K Extracting clinically relevant data from finite element simulations Clin Biomech 2005 20 451 454 10.1016/j.clinbiomech.2005.01.010 15836931

[B43] Taylor WR Heller MO Bergmann G Duda GN Tibio-femoral loading during human gait and stair climbing J Orthop Res 2004 22 625 632 10.1016/j.orthres.2003.09.003 15099644

[B44] Zheng N Fleisig GS Escamilla RF Barrentine SW An analytical model of the knee for estimation of internal forces during exercise J Biomech 1998 31 963 967 10.1016/S0021-9290(98)00056-6 9840764

[B45] Guevarra Y Implants for surgery: Wear of total knee joint prostheses 1999 London: International Organization for Standardization

